# Percutaneous management of chronic total occlusion of the portal vein: a retrospective analysis of technical aspects and outcomes

**DOI:** 10.1186/s42155-024-00496-w

**Published:** 2024-11-23

**Authors:** Ludovico Dulcetta, Paolo Marra, Riccardo Muglia, Francesco Saverio Carbone, Mauro Viganò, Angelo Di Giorgio, Lorenzo D’Antiga, Stefano Fagiuoli, Sandro Sironi

**Affiliations:** 1 Department of Radiology, ASST Papa Giovanni XXIII Hospital, Piazza OMS, 1, Bergamo, 24127 Italy; 2 Gastroenterology Hepatology and Transplantation Unit, ASST Papa Giovanni XXIII, Piazza OMS, 1, Bergamo, 24127 Italy; 3 Department of Paediatric Hepatology, Gastroenterology and Transplantation, ASST Papa Giovanni XXIII Hospital, Piazza OMS, 1, Bergamo, 24127 Italy; 4 School of Medicine and Surgery, University of Milano-Bicocca, Piazza Dell’Ateneo Nuovo, 1, Milan, 20126 Italy

**Keywords:** Liver transplantation, Cavernous transformation of the portal vein, Portal hypertension, Angioplasty, Stents, TIPS

## Abstract

**Background:**

Chronic total occlusion (CTO) of the portal vein is one of the main causes of portal hypertension, which may result in life-threatening complications often managed by interventional radiology (IR). The aim of this study is to report the innovative experience with percutaneous revascularization therapy in the management of portal vein CTO in paediatric and adult patients.

**Materials and methods:**

From January 2020 to December 2023 consecutive paediatric and adult patients with severe portal hypertension resulting from portal vein CTO who underwent attempts at percutaneous recanalization were retrospectively reviewed. Technical aspects including the percutaneous approach, portal vein stenting, transjugular intrahepatic portosystemic shunt (TIPS) creation, varices embolization and clinical outcomes including adverse events and control of portal hypertension were analyzed. Technical success was defined as at least partial restoration of the portal vein patency at the final angiogram. Clinical success was defined as the improvement of clinical-laboratory signs of portal hypertension and control for variceal bleeding.

**Results:**

Fifteen patients (median age = 21 years, range = 59 years; 10 males; 5 children) with portal vein CTO underwent a total of 25 percutaneous revascularization procedures. Nine patients (60%; 5 children, 4 adults) were liver transplant recipients. All patients except one had cavernous transformation of the extra-hepatic portal vein, involving the spleno-mesenteric confluence in 5 cases. Technical success was achieved in 13/15 (87%) patients of whom 8 had portal revascularization through the placement of an extra-hepatic stent; indeed, in six cases, a TIPS was performed to achieve sustained portal vein patency. Embolization of varices and/or cavernoma was performed in 12 patients. Adverse events occurred in 2/15 (splenic artery perforation and hemoperitoneum, one each) managed without sequelae. Technical success led to clinical success in all the 13/15 (87%) cases, with a median follow-up of 20 months (IQR 4–34 months).

**Conclusion:**

CTO can be managed effectively by interventional radiology. Restored portal flow physiology alone is possible in most patients, while TIPS may be required in a small proportion of them, to prolong portal vein patency and control portal hypertension.

## Background

Chronic total occlusion (CTO) of the portal vein resulting from portal vein thrombosis, either primary or secondary to an underlying chronic liver disease, is a potentially life-threatening condition. It represents the most common cause of prehepatic portal hypertension and is associated with gastrointestinal bleeding, portal cholangiopathy, hypersplenism and ascites [1–11].

The underlying etiology of portal vein CTO remains unclear in up to 50% of children and adults [2].

Portal vein CTO also frequently occurs as a complication of liver transplantation (LT), and it is associated with high mortality and graft loss. Although the incidence is very low in adults [12], portal vein thrombosis leads to impaired 5-year graft survival [12, 13].

In patients with portal vein CTO after LT, ischemic damage to the hepatic parenchyma and ischemic biliopathy may eventually cause biliary cirrhosis of the graft, even in the presence of a cavernoma. Furthermore, extensive porto-systemic shunting may lead to hepatic encephalopathy, hepatopulmonary syndrome or porto-pulmonary hypertension. For all the above-mentioned clinical implications, appropriate management of portal vein CTO is paramount [12–14].

The aim of this study is to report the experience of a tertiary referral center for paediatric and adult liver disease and transplantation in portal vein recanalization (PVR) of portal vein CTO, with focus on technical aspects and clinical outcomes.

## Materials and methods

### Study design

This retrospective cohort study includes consecutive paediatric (< 18 years old) and adult patients with portal hypertension resulting from portal vein CTO who underwent attempts at PVR from January 2020 to December 2023. Patients’ data including clinical, biochemical, and imaging findings, were retrospectively reviewed through interrogation of medical records and anonymized for the analysis.

Inclusion criteria were an imaging diagnosis of portal vein CTO and clinical signs of portal hypertension, namely hypersplenism (defined by splenomegaly and platelet count below the normal values), any grade of gastrointestinal varices and/or ascites. Exclusion criteria was an imaging diagnostic of acute or subacute portal vein thrombosis, previous splenectomy, and absence of obvious signs of portal hypertension.

This research retrieved a total of 15 consecutive patients (median age = 21 years, range = 59 years; 10 males; 10 adults) who underwent a total of 25 percutaneous procedures, from January 2020 to December 2023.

All cases were discussed in the liver multidisciplinary team, including interventional radiologists, adult and or paediatric hepatologists and LT surgeons. All patients underwent screening for hematologic prothrombotic disorders.

Written informed consent was obtained for every diagnostic and interventional radiology procedure from adult patients and from all the patients’ parents (both mother and father) or legal guardians. The Ethical Committee of Bergamo authorized this retrospective study (Portal01; N.92/21) that was conducted in respect of the ethical standards laid down in the 1964 Declaration of Helsinki.

### Diagnosis

Diagnosis of portal vein CTO was based on the findings of imaging techniques including color-Doppler ultrasound (CDUS), CT angiography (CTA), and/or MR angiography (MRA). CTO of the portal vein, portal vein CTO extension and cavernous transformation assessment were established according to previous reports [11].

### Percutaneous PVR

All procedures were performed under general anesthesia by dedicated anesthesiologists for the paediatric population.

Interventional radiologists with at least 5 years of experience in hepatobiliary interventions performed the procedures under fluoroscopy and digital subtraction angiography (DSA) (Allura Xper FD20; Philips Healthcare, Best, the Netherlands) and intra-procedural CDUS (Affiniti 70G; Philips Healthcare, Best, the Netherlands) guidance.

Based on the extension of CTO and pre-procedural mapping, the most favorable access was chosen among the retrograde transhepatic and the antegrade transplenic route. The transhepatic access was favored whenever native intrahepatic portal branches could be visualized with CDUS. The transplenic access was selected as secondary option whenever the transhepatic one was not feasible, with absolute (splenectomy) or relative (ascites; micro-polysplenia and splenic vein thrombosis) contraindications. In case of ascites the placement of a pre-operative peritoneal drainage was considered; in case of polysplenia and splenic vein thrombosis attempts at catheterization were performed provided that intrasplenic patent vessels were judged targetable on a pre-operative explorative CDUS examination. The transjugular access was performed based on intra- or post-procedural findings if transjugular intrahepatic porto-systemic shunt (TIPS) creation was deemed necessary for the following reasons: lack of hepatopetal portal flow after PVR; persistent abnormal (> 12 mmHg) porto-systemic gradient after successful PVR; proved cirrhosis.

Percutaneous accesses were performed and managed as previously described [15]. CTO recanalization was attempted under ultrasound guidance with dedicated devices including hydrophilic 0.014″ guidewires (Abbott, Command), 1.9 French microcatheters (Terumo, Progreat Lambda), PTA microcatheters (Boston Scientific, Sterling) and snares (Andramed, Andrasnare). After successful PVR, angioplasty was performed with non-compliant balloon catheters (Boston Scientific, Mustang) of increasing sizes up to 12 mm in the extrahepatic portal tract. Indications to portal vein stenting included intra-operative residual stenosis or recurrent stenosis during follow-up. The main criteria that were considered in the choice of the type of metal stent were the presence of branch vessels, the size of the target vessel and the potential of patient’s growth. Bare-metal stenting (Abbott, Absolute Pro and Omnilink Elite) was considered in the first instance to preserve patency of lateral branches. Covered stenting (Gore, Viabahn VBX) was reserved for secondary treatment of in-stent occlusions. Balloon-expandable stents were preferred for paediatric patients with a potential of growth since they are suitable for post-dilation up to 10–30% of the nominal size.

Persistent opacification of varices and cavernoma after PVR with significant flow steal phenomenon was considered an absolute indication to embolization which was performed either with coils (Cook, MREye) and/or cyanoacrylate (GEM, Glubran 2). Only coils were employed when leak of liquid embolic was anticipated, and the target of embolization was the complete disappearance of collateral flow. If deemed necessary, TIPS was accomplished with a modified “gun-sight” technique [16]. Namely, through the transhepatic or transplenic access a snare catheter was navigated into the right portal vein and targeted under ultrasound guidance with a 22-gauge Chiba needle that was advanced coaxially up to the hepatic vein.

From the transjugular access the transhepatic wire advanced through the Chiba needle was snared to achieve a “through and through’’ access. The transplenic or transhepatic snare was then pulled to allow navigation of the portal vein from the transjugular access. Finally, TIPS creation (Gore, Viatorr) was completed in the standard fashion.

### Outcome measures

Technical success was defined the complete or at least partial flow restoration in the portal vein at the end of the procedure. Clinical success was represented by clinical control of portal hypertension. Primary technical success was defined as patency of the portal vein after the first procedure; secondary technical success was defined as patency of portal vein after subsequent PVR attempts.

Secondary endpoints were sustained patency of the portal vein during follow-up and safety of the procedures in terms of complications and adverse events which were graded according to the CIRSE Quality Assurance Document and Standards for Classification of Complications [17].

Portal vein patency after PVR was assessed by CDUS, routinely performed after 24 h, 1 week and 1 month. In the absence of stenosis or thrombosis recurrence, further follow-up continued every 6–12 months as per the institutional protocol. CTA examinations were performed only if clinically indicated.

### Statistical analysis

Continuous data are presented as the medians ± interquartile range (IQR); categorical data as counts (percentage). Descriptive statistics were calculated in Microsoft Excel 2016 (Microsoft Corporation, Redmond, WA, USA). Descriptive and analytic statistics were calculated using IBM SPSS Statistics Version 27.0 (IBM Corp., Armonk, NY).

## Results

### Study sample

Patients’ data at baseline are outlined in Table 1.

**Table 1:** Patients’data, portal vein thrombosis and baseline clinical features

**N.**	**Age (y)**	**Gender**	**OLT**	**Liver histology**	**Baseline imaging findings**	**Portal Cavernoma**	**Portal Hypertension**	**Clinical-laboratory signs**	**FU time (m)**	
1	17	M	Yes	No cirrhosis	MPV thrombosis	Yes	Yes	Hypersplenism, splenomegaly	60	
2	7	M	Yes	No cirrhosis	MPV thrombosis	No	Yes	Hypersplenism, splenomegaly	36	
3	16	M	Yes	No cirrhosis	MPV thrombosis	Yes	Yes	Hypersplenism, splenomegaly	39	
4	63	M	Yes	n/a	MPV, SMV and SV confluence thrombosis	Yes	Yes	Variceal bleeding	34	
5	45	F	No	n/a	MPV, SMV and SV confluence thrombosis	Yes	Yes	Variceal bleeding, congestive gastropathy	8	
6	16	M	Yes	No cirrhosis	MPV thrombosis	Yes	Yes	Hypersplenism, splenomegaly, congestive gastropathy	30	
7	10	F	Yes	No cirrhosis	MPV thrombosis	Yes	Yes	Variceal bleeding, congestive gastropathy	31	
8	21	F	Yes	No cirrhosis	MPV thrombosis	Yes	Yes	Variceal bleeding, splanchnic aneurysm,	30	
9	21	M	Yes	Mild liver fibrosis	MPV thrombosis	Yes	Yes	hypersplenism, splenomegaly	20	
10	66	M	No	Mild liver fibrosis	MPV thrombosis	Yes	Yes	Variceal bleeding, congestive gastropathy	4	
11	62	M	No	Cirrhosis	MPV, SMV and SV confluence thrombosis	Yes	Yes	Variceal bleeding, congestive gastropathy	18	
12	21	M	No	n/a	MPV thrombosis	Yes	Yes	Variceal bleeding, hypersplenism, splenomegaly	5	
13	61	M	No	n/a	MPV, SMV and SV confluence thrombosis	Yes	Yes	Variceal bleeding, hypersplenism, splenomegaly	3	
14	54	F	No	n/a	MPV, SMV and SV confluence thrombosis	Yes	Yes	Variceal bleeding, hypersplenism, congestive gastropathy	3	
15	19	F	Yes	n/a	MPV thrombosis	Yes	Yes	Variceal bleeding, hypersplenism, splenomegaly	3	

Among 15 patients (median age = 21 years, range = 59 years; 10 males; 10 adults), 5 were children (median age = 16 years, range = 10 years; 4 males).

The majority of patients (9/15, 60%) were LT recipients (5 children, 4 adults). Among them, 6/9 (67%) received a left lateral split graft, and 3/9 (33%) received a whole liver, all from deceased donors. The most frequent indication for LT (6/9, 67%) was biliary atresia; cryptogenic cirrhosis, end-stage cirrhosis and Alagille syndrome were respectively the remaining indication for LT.

All patients had CTO of the main portal vein, either with (*n* = 5) or without (*n* = 10) CTO of the superior mesenteric vein and/or the splenic vein. Cavernous transformation was noted in 14/15 (93%) patients: in 7/14 (50%) cases cavernous transformation of intra- and extrahepatic portal vein was found, in 7/14 (50%) cases it was limited to the extrahepatic portal tract. In 12/15 (80%) patients a main portal vein fibrotic vestige was seen.

Liver histological data were available for 9 out of 15 patients: six were non-cirrhotic (5 children, 1 adult), two had mild liver fibrosis, and one was cirrhotic. Of those patients without liver histopathology, none presented clinical or radiological signs of cirrhosis.

No systemic risk factors for thrombosis were identified in this population study.

At the time of treatment, all patients presented clinical signs of portal hypertension: 11 patients had a recent history of recurrent variceal bleeding; 9 had hypersplenism with severe thrombocytopenia. One patient had multiple splanchnic arterial aneurysms resulting from hyperdynamic circulation.

The median delay from CTO diagnosis to treatment was 13 months (IQR = 5–131 months): 3 months (IQR = 1—107,5 months) in paediatric population, 18,5 months (IQR = 8,75 – 149,5 months) in adult one.

### Imaging diagnosis

In 7 patients, the first diagnosis of portal vein CTO was made with CDUS; in 7 cases portal vein CTO was first detected with CTA, while in 1 patient, diagnosis of portal vein CTO was demonstrated with MRA. All patients required further pre-procedural assessment with CTA.

### Technical and clinical outcome

Procedural details and outcomes are reported in Table 2.

**Table 2:** Procedural details and clinical outcomes

**N.**	**Delay time (m)**	**Primary technical success**	**Reintervention**	**Second technical success**	**Recurrence/persistence**	**TIPS placement**	**Portal stent placement**	**Variceal embolization**	**Complications**	**Overall clinical success**	
1	3	No	Yes	Yes	No	No	Yes	Yes	None	Yes	
2	1	Yes	No	-	No	No	Yes	No	None	Yes	
3	84	Yes	Yes	Yes	No	No	Yes	Yes	None	Yes	
4	13	No	Yes	Yes	No	Yes	Yes	Yes	None	Yes	
5	5	No	No	-	Yes	No	No	No	None	No	
6	131	Yes	No	-	No	No	No	Yes	None	Yes	
7	1	Yes	Yes	Yes	No	No	No	Yes	None	Yes	
8	145	No	Yes	Yes	Yes	Yes	Yes	Yes	None	Yes	
9	122	No	Yes	Yes	No	Yes	Yes	Yes	None	Yes	
10	163	Yes	No	-	No	Yes	Yes	Yes	None	Yes	
11	24	Yes	Yes	Yes	No	Yes	No	Yes	None	Yes	
12	5	Yes	No	-	No	No	No	Yes	Mildperisplenicbleeding	Yes	
13	11	No	No	-	Yes	No	Yes	No	Perforationofsplenicartery	No	
14	10	Yes	No	-	No	Yes	No	Yes	None	Yes	
15	168	Yes	No	-	No	No	Yes	Yes	None	Yes	

**Fig. 1:**
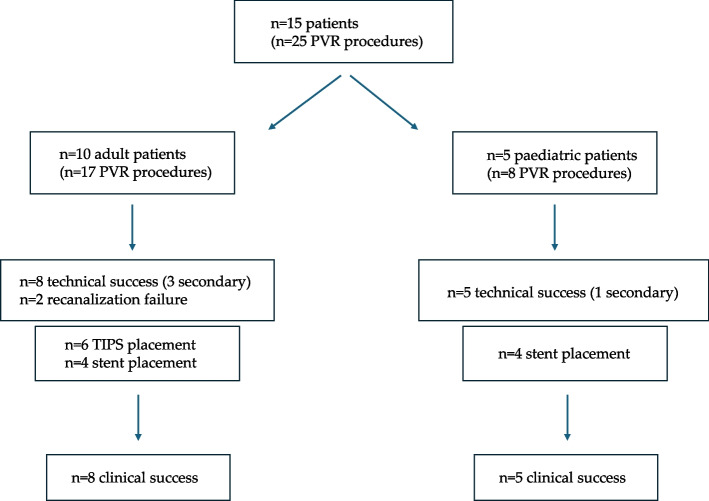
Algorithm of the study population

A total of 25 percutaneous transhepatic, transplenic and/or transjugular procedures were carried out. Figure 1 summarizes the results.

**Fig. 2:**
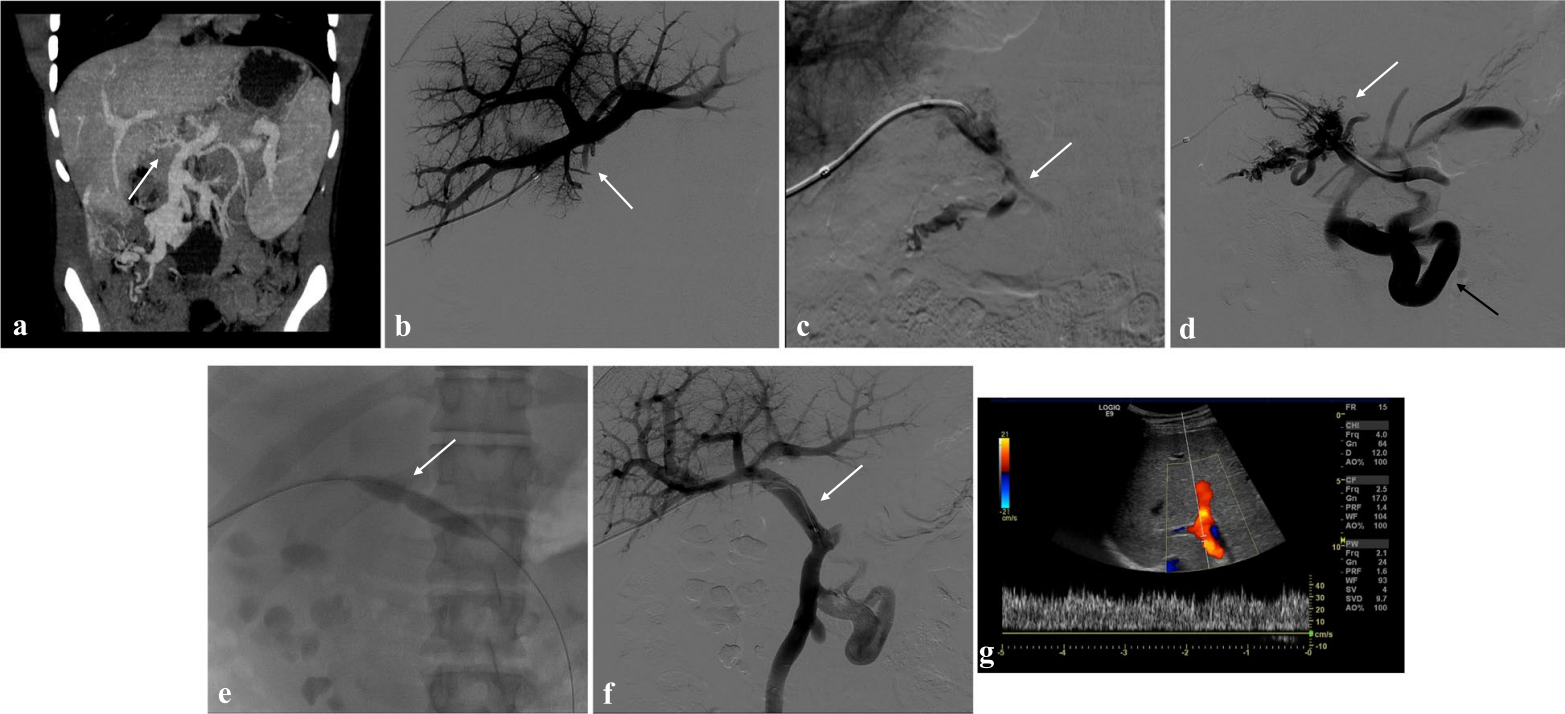
Portal vein CTO in a 16-year-old boy with a history of portal hypertension 15 years after liver transplantation (whole graft) for Alagille syndrome. **a** Portal phase coronal CT image shows the complete portal vein thrombosis involving the portal extrahepatic trunk with cavernous transformation (arrow). **b** Anteroposterior view of a percutaneous transhepatic portography image shows a regular representation of the intrahepatic portal branches. Complete occlusion of the extrahepatic portal vein at the hepatic hilum (arrow) is demonstrated. **c** Portography image shows the opacification of a very tiny vascular structure (arrow) which represents the main portal vein fibrotic vestige. **d** Inferior mesenteric portography image obtained through a 5Fr catheter which was advanced across the obstructed tract confirms the total occlusion of the main portal vein, with opacification of enlarged porto-mesenteric varices (black arrow) and portal cavernoma (white arrow). **e** Fluoroscopic image shows angioplasty of the main portal vein, performed through a 12-mm non-compliant balloon catheter. The focal notch (arrow) represents the tight anastomotic stenosis that probably led to secondary thrombosis. **f** Control portography shows a re-expanded extrahepatic portal vein (arrow) with normal opacification of intrahepatic portal branches. **g** Color-Doppler Ultrasound follow-up image shows regular hepatopetal flow of the main portal vein

Primary technical success was achieved in 9 of 15 patients (60%) (Fig. 2). Secondary technical success was achieved in 4 of the primarily failed 6 patients (67%). Two PVR attempts failed. In one case (patients #8), four PVR attempts were accomplished in a young adult with split liver graft received during the neonatal age with a 20-year history of portal vein CTO and portal hypertension. In the first procedure portal vein was recanalized through a percutaneous transhepatic access and a bare metal stent was placed in the main portal vein to maintain the patency. After the evidence of acute portal vein thrombosis including stent thrombosis, a combined transplenic, transhepatic and transjugular access were performed to recanalize the portal vein’s stent and to create a TIPS. A third procedure was performed due to recurrence of partial portal stent thrombosis, managed with in-stent covered-stenting. Finally, a fourth transjugular aspiration thrombectomy procedure was performed due to portal vein thrombosis recurrence. Eventually, portal vein stent and TIPS occlusion was noted at the last follow-up, but no further PVR interventions were proposed since portal hypertension was compensated through a spontaneous splenorenal shunt. Platelet count dramatically improved after partial splenic parenchyma and splenic artery aneurysms embolization.

**Fig. 3:**
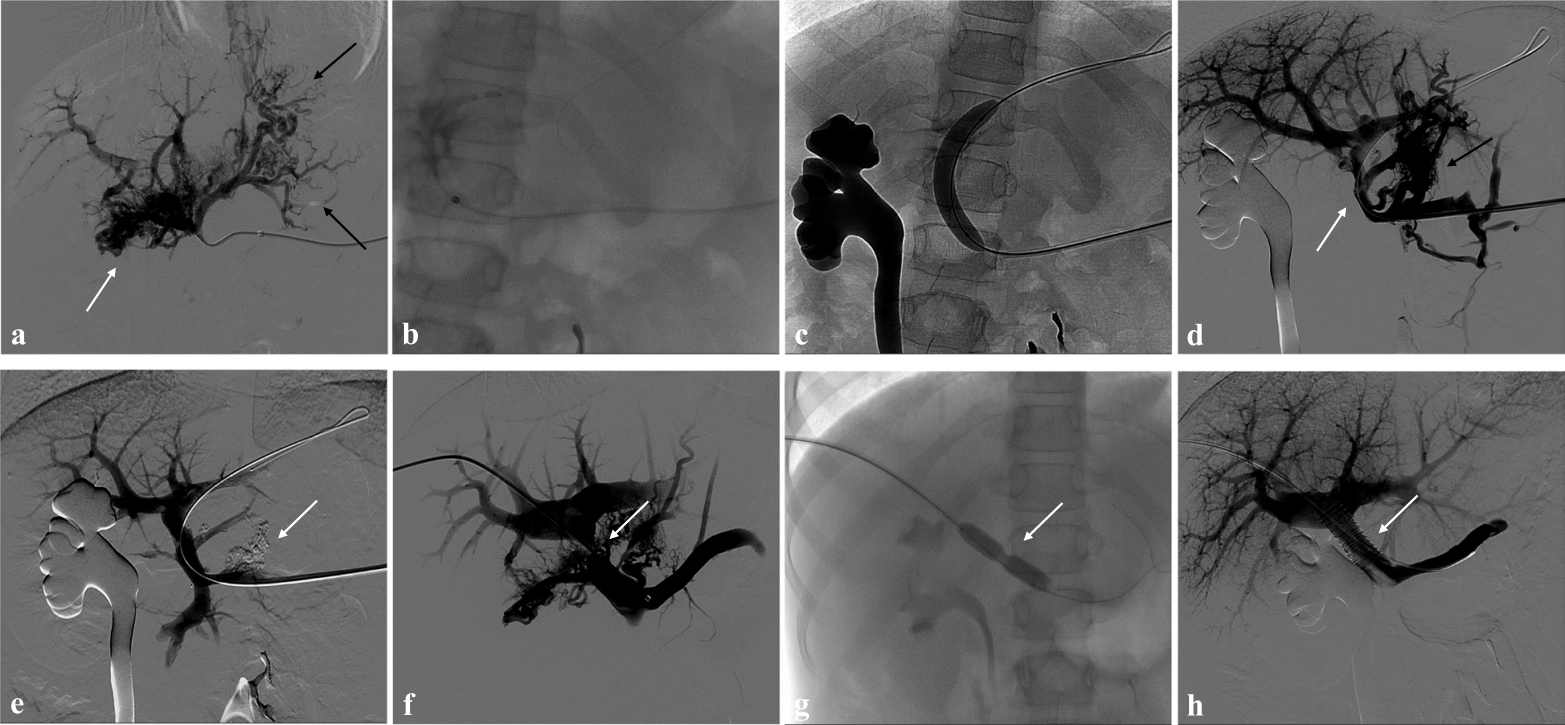
Portal vein CTO in a 10-year-old girl with a history of portal hypertension 10 years after liver transplantation (whole graft) for biliary atresia. **a** Percutaneous transplenic portography shows total occlusion of the main portal vein, with opacification of several portosystemic varices (black arrows) and portal cavernoma (white arrow). **b**, **c** Fluroscopic image shows angioplasty of the main portal vein after its successful catheterization, performed respectively through a 3-mm non-compliant balloon microcatheter and a 10-mm non-compliant balloon catheter. **d** Control portography shows a recanalized extrahepatic portal vein (white arrow) with normal opacification of intrahepatic portal branches and persistent opacification of portosystemic varices (black arrow). **e** Control portography after portosystemic varices embolization performed using a mixture of N-butyl cyanoacrylate and iodized oil shows an improved hepatopetal portal flow of the extrahepatic portal vein. Note subtraction artifacts (arrow) representing lipiodol in the varices. **f** Percutaneous transhepatic portography performed 9 months after the first angioplasty treatment shows residual stenosis of the revascularized extrahepatic portal vein (arrow), with opacification of portal cavernoma and portosystemic varices. **g** Fluroscopic image shows angioplasty of the main portal vein performed through a 12-mm non-compliant balloon catheter. The focal notch (arrow) represents the tight anastomotic stenosis. **h** Final portogram performed after deployment of a 10-mm self-expandable bare metal stent (arrow) to treat residual stenosis of the extrahepatic portal vein; the main portal vein is now regularly opacified with adequate size and both intrahepatic portal branches present hepatopetal flow

Additional extrahepatic portal vein stents were deployed in 8 patients: in patients #8, #9, #12 and #15 a bare metal stent was placed across the recanalized portal vein in the same PVR procedure; patient #2 underwent primary stent grafting. In patient #3 recurrence of complete extrahepatic portal vein CTO 1 year after the first angioplasty treatment required a bare metal stent placement for an evident residual stenosis; in patients #1 and #7 recalcitrant stenosis of the revascularized extrahepatic portal vein respectively 3 and 9 months after successful PVR required bare metal stenting (Fig. 3). Additionally, in six cases (40%), all adults, a TIPS was performed to achieve sustained portal vein patency. In all patients in the TIPS subgroup, self-expandable polytetrafluoroethylene -ePTFE- covered stents were used.

**Fig. 4:**
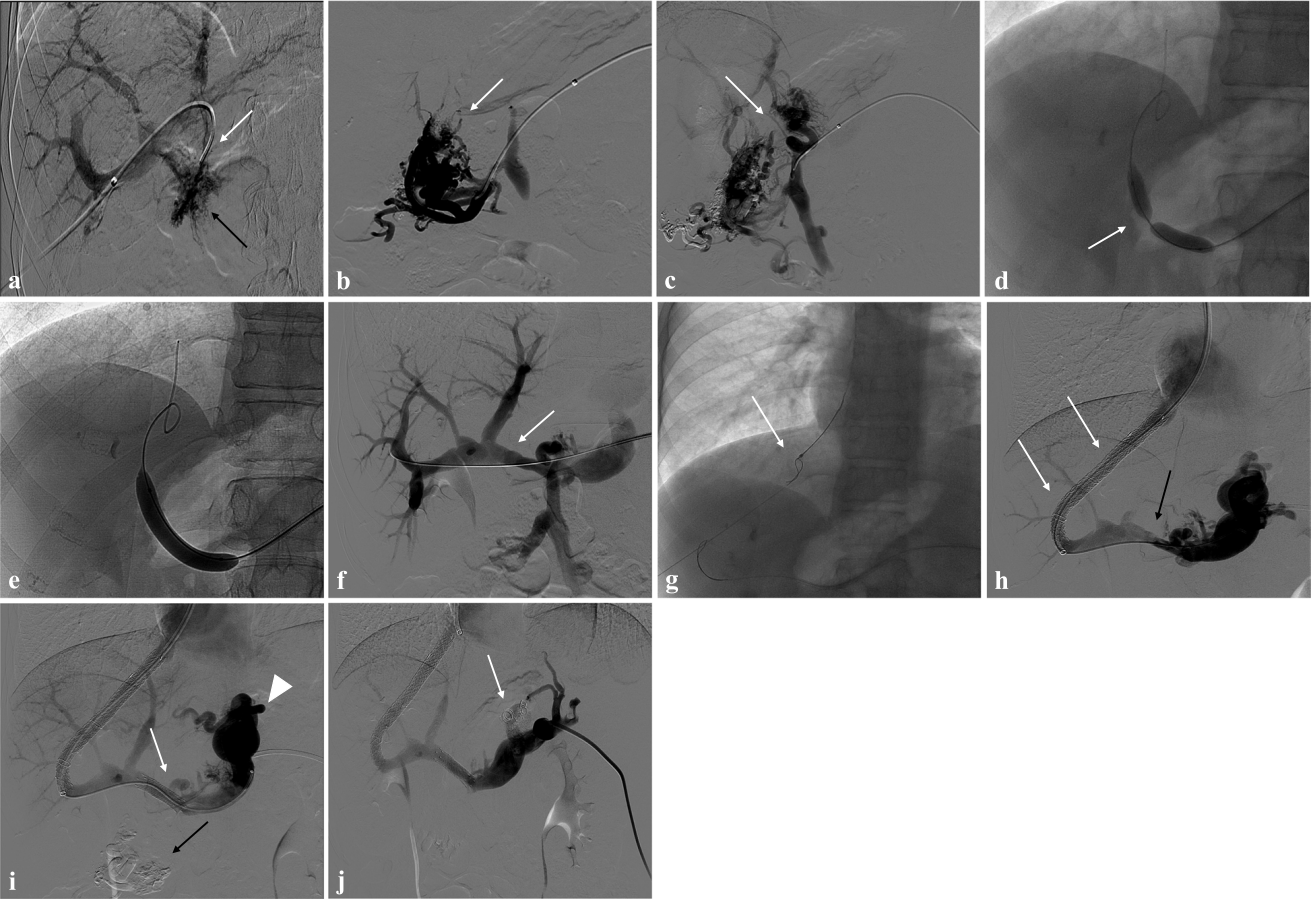
Portal vein CTO in a 21-year-old boy with a history of portal hypertension 9 years after liver transplantation (whole graft) for biliary atresia. **a** Percutaneous transhepatic portography shows complete extrahepatic portal vein thrombosis (white arrow), with opacification of tiny irregular portal cavernomatous vessels at hepatic hilum (black arrow). **b** Percutaneous transplenic anterograde portography shows complete extrahepatic portal vein thrombosis and cavernous transformation (arrow). **c** The tiny vestige of the main portal trunk was detected (arrow) on the transplenic superior mesenteric vein portography. **d**,** e** Fluoroscopic image shows angioplasty of the main portal vein, performed through a 12-mm non-compliant balloon catheter. The focal notch (arrow) representing the tight anastomotic stenosis that probably led to secondary thrombosis was resolved after high-pressure inflation of the non-compliant 12-mm balloon catheter. **f** Control portography shows an expanded main portal vein with improved hepatopetal portal flow, but still with irregular profiles (arrow). **g** Through a hybrid transhepatic and transjugular approach under combined ultrasound and fluoroscopic guidance using a modified “gun-sight” technique, the connection between the intrahepatic portal vein and the vena cava was created advancing a 0.018″ micro guidewire into the right atrium. The wire was snared through the right transjugular access providing a through-and-through access (arrow) for transjugular intrahepatic portosystemic shunt (TIPS) placement. **h** Portography image shows the deployment of two imbricated Viatorr stents to create the TIPS (arrows), with regular intrastent opacification. Despite previous angioplasty, a prestenotic aspect of the main portal vein is seen (black arrow). **i** Portography image shows a self-expandable 9-mm metallic stent placed to cover the main portal vein trunk stenosis (white arrow). Note subtraction artifacts (black arrow) representing the result of variceal embolization using a mixture of N-butyl cyanoacrylate and iodized oil and metallic coils. Large gastric varices were seen (arrowhead). **j** Control portography shows regular portal vein axis opacification with TIPS patency and preserved opacification of intrahepatic portal branches; gastric varices disappeared after their embolization (arrow)

The transhepatic puncture of an intrahepatic portal branch was performed in 12/25 (48%) procedures. The transplenic access was performed in 9/25 (36%) procedures. A combined transhepatic and transplenic approach was performed in 4/25 (16%) procedure. The transjugular approach was used in all cases of TIPS placement (*n* = 6) (Fig. 4).

Clinical success during a median follow-up of 20 months (IQR 4—34 months) was achieved in all technically successful cases. Variceal embolization was performed in 12 cases. None of the patients with a history of variceal hemorrhage rebled. Disappearance of high-risk gastroesophageal varices was documented in all technically successful cases at endoscopic examinations performed during the follow-up. In those who had a successful PVR, a significant improvement in serum levels of platelet count was found at three-month post-procedure follow-up. In patients with primary patency at 3 months, platelet count increased from a median of 76 × 10^9^L (48,5—134 × 10^9^L) to a median of 114,5 × 10^9^L (75—169,75 × 10^9^L; *p* < 0.05).

As per institutional protocol, all patients received a therapeutic dose of low-molecular-weight heparin for 6 months after successful PVR procedures. Afterwards, anticoagulation was withheld.

Complications (grade 3) occurred in 2/15 (13%) patients: one splenic artery perforation occurred during a transplenic procedure. The splenic vein was also thrombosed, and during recanalization attempts, the guidewire inadvertently perforated the splenic artery. Angiography following catheterization revealed opacification of the aorta. The catheter was then retracted to the proximal segment of the splenic artery, which was occluded using coils. No contrast extravasation or peritoneal fluid accumulation was observed. The parenchymal segment was subsequently embolized with glue. The patient experienced no clinical symptoms or long-term sequelae. One instance of periprocedural capsular splenic bleeding resulted in hemoperitoneum, which was conservatively managed without clinical consequences.

### Imaging follow-up

In 12/13 (92%) technically successful cases, portal vein remained patent throughout a median follow-up of 20 months (IQR 4—34 months). One-month post-procedure follow-up imaging showed patent portal vein with excellent flow in all patients. TIPS thrombosis was observed in 1 case.

## Discussion

Here we report a series of patients affected by portal hypertension resulting from CTO of the portal vein. All of them presented with severe complications, the worst of which was variceal hemorrhage. Portal vein CTO may cause severe hemorrhage and multi-organ failure [18] in cirrhotic and non-cirrhotic patients but can also complicate LT [19]. In our series the prevalence of non-cirrhotic and liver-transplanted patients was balanced with that of those affected by chronic liver disease and with overt cirrhosis. However, patients with cirrhosis may represent a subgroup for whom the endovascular management with PVR needs associated treatments like variceal embolization and TIPS creation, to achieve sustained portal vein patency and clinical efficacy. The main finding of our study was that at least half of patients manifesting portal vein CTO may benefit from percutaneous recanalization without the need of TIPS creation. Therefore, we strongly recommend considering PVR as the primary, stand-alone objective of the procedure. If PVR is successful, stenting of the previously occluded portal vein may be warranted in cases of stricture or recurrent thrombosis, as observed in half of the patients in our series.

On the other hand, after PVR some patients may require primary or secondary TIPS if they present impaired portal flow, not only due to cirrhosis-related increased resistance, but also due to long-standing portal vein occlusion with extensive porto-systemic shunts and large cavernomas leading to flow steal phenomenon.

Unfortunately, no preoperative imaging features were found to be able to predict the need for stenting, TIPS creation and/or varices embolization in our study.

Most of our patients were liver-transplanted without overt cirrhosis. Portal vein CTO is a rare complication of LT, and it is associated with high mortality and graft loss. Although the incidence is very low in adults, it leads to a reduction in 5-year graft survival when compared with LT recipients without portal vein complications [13]. Compared to adults, paediatric patients are at greater risk of developing post-transplant portal vein CTO with an incidence rate raising up to 3–14% after living-donor LT compared with 2–3% after a deceased-donor LT [20]. In our series, 5/8 (63%) LT recipients were paediatric patients at the PVR’s time. In patients with LT, PVR with or without TIPS or portal stent placement has proven to be an advantageous treatment option for post-transplant portal vein CTO because it not only re-establishes portal flow but also treats hypersplenism secondary to portal hypertension [21, 22].

Despite the meso-Rex bypass is considered the treatment of choice for extrahepatic portal vein CTO given its unique ability to re-establish physiological hepatic portal venous blood flow [23], PVR may be considered equally effective and less invasive as demonstrated in our series.

However, the role of percutaneous PVR in chronic extrahepatic portal vein CTO is not yet established, especially in paediatric patients with long-term occlusion [24].

Few paediatric studies in the literature have reported successful PVR in paediatric patients after LT, paving the way for this procedure to be adopted as a feasible alternative to the standard surgical option in experienced centers [20, 22, 25, 26].

In the subgroups of paediatric patients (*n* = 5) of this study, percutaneous transhepatic or transplenic PVR was technically and clinically successful in all cases. No procedure-related complications were observed during the follow-up period. Furthermore, none of the paediatric patients required TIPS placement as PVR and varices or cavernoma embolization were enough to restore an adequate portal flow to the liver. Despite the small numbers of patients, in our experience percutaneous PVR was effective, and it allowed to avoid the surgical risks of the meso-Rex operation. More data is required to affirm the role of the endovascular approach compared to the standard of practice represented by surgery. Indeed, after successful PVR, many studies reported higher rates of portal vein CTO recurrence [18, 27, 28].

In our series sustained portal vein patency in technically successful cases was 92% (12 of 13 patients) at the last follow-up. As observed by Klinger et al. [29], one reason for these favorable results may be the embolization of large portosystemic collaterals to prevent the flow steal phenomenon. No consensus exists on the benefit of variceal embolization in patients with portal vein CTO. In the treatment of late onset portal vein CTO with clinical signs of portal hypertension, decreased portal flow due to severe portosystemic shunt may contribute to recurrent portal vein thrombosis itself, and restoration of the portal flow with simultaneous embolization of portosystemic shunts may allow to achieve a sustained patency of the portal vein [25, 30, 31]. Moreover, no consensus exists in the timing of variceal embolization. In our experience, all cases of variceal embolization were performed during the PVR procedure. Other authors did not perform variceal embolization at the time of PVR procedure and TIPS placement, but after 1 month. The reason relied on the aim to maintain the mesenteric outflow through the varices in case of re-thrombosis of portal vein and to limit radiation exposure and contrast loads [32].

One argument of debate is the choice between PVR alone or associated with TIPS creation. As suggested by Marot et al. [33] the combined approach should be aimed at alleviating portal hypertension as much as possible. According to this study, TIPS would likely be useful when portal hypertension is also related to an intrahepatic block of the portal circulation. However, as intrahepatic pressures are normal in most patients with extrahepatic portal vein obstruction, the benefit of associating TIPS to PVR remains unclear and should be evaluated on an individual basis [1].

In our experience, we associated TIPS to PVR in half of the patients, all of whom were adults. In this subgroup of patients, TIPS placement was deemed necessary after PVR due to a persistently increased portosystemic gradient suggesting unresolved portal hypertension. Interestingly, no correlation between cirrhosis and TIPS requirement was found. TIPS may improve portal venous hemodynamics and induce flow-enabled clot dissolution. To this end, in a previously reported series, portal venous flow was increased more than fivefold after TIPS, and TIPS produced a sustained portal vein patency in four LT candidates with partial portal vein thrombosis [34].

To summarize, according to our protocol, following a successful PVR, we always recommend performing variceal and cavernoma embolization if persistent opacification is observed. Additionally, we advise considering primary TIPS creation only in specific situations: namely, in cases of histology-confirmed cirrhosis and/or inadequate hepatopetal flow despite a patent, stenosis-free portal vein and complete variceal/cavernoma embolization. This approach is essential for preventing long-term complications from portosystemic shunting, particularly in young patients who are unlikely candidates for future liver transplants.

Our results demonstrate safety and efficacy of PVR-TIPS. Of note, we described a modified technique for TIPS creation taking advantage of the percutaneous approach with the so called “gun-sight” technique [16]. This modality allowed us to accomplish TIPS creation even in patients with unusual anatomic conditions like those with split liver grafts. The remaining half of patients who underwent PVR alone were all paediatrics. Notably, no recurrence of portal vein CTO was noted at imaging examination during follow-up period in this subgroup of population study, demonstrating that TIPS creation may be not necessary with PVR.

Another argument of discussion regards the median delay from diagnosis of portal vein CTO to treatment: in our study the median time delay was 13 months (IQR = 5–131 months). In one third of the patients, more than 10 years elapsed between diagnosis and successful treatment, emphasizing that there is no theoretical time threshold beyond which the PVR procedure cannot be attempted.

Feasibility of PVR is often not predictable using preoperative imaging. As shown in a study [33], preoperative imaging lacked diagnostic accuracy in predicting when PVR was not feasible in portal vein CTO. Wedge hepatic venography should be performed upfront whenever no intrahepatic portal branches are visualized to decide if PVR should be attempted. Based on our experience an -even partial- intrahepatic portal vein visualization represents an indication to attempt PVR.

Similarly to one study [29] but in contrast with others [35, 36], the majority of our patients showed main portal vein replacement with a fibrotic cord (grade IV according to Qi et al. portal vein CTO’s grading classification) [37] originating along the superior margin of the portal confluence. However, when cavernoma is present, the origin of the native portal vein may be difficult to identify and catheterize. In these cases, contrast injection may determine faint opacification of the fibrotic cord of the chronically occluded portal vein. According to our experience, PVR must be attempted whenever this tiny vestige of the main portal trunk is detected.

As shown by Salem et al. [38], most notably for LT patients, PVR-TIPS frequently transformed the portal vein from a small, virtually nonexistent fibrotic cord to a vein of normal diameter without thickening, induration or scarring which was readily sewn to the donor portal vein without difficulty. This operative finding was confirmed in 18 transplanted patients after PVR-TIPS.

Consensus lacks also for the optimal indication for portal vein stenting. Six of our successfully recanalized patients received metal stents that were all bare-metal except one. No criteria exist to favor the use of bare-metal stent or grafts, which are significantly more expensive.

Criteria exist to indicate stenting, namely a suboptimal angioplasty result with residual pressure gradient > 5 mm Hg or elastic recoil of the stricture greater than 50%, vessel dissection, recurrence of portal vein stenosis within 3–6 months and portal vein kinking [25, 26, 39]. Moreover, portal vein stenting seems to be safe and effective for the treatment of post-LT portal vein occlusion with underlying anastomotic obstruction in paediatric recipients. The intermediate-term portal vein patency rates after stent placement are excellent, up to 100% [40–42].

These patency rates are superior to those previously reported with balloon angioplasty alone, ranging from 27 to 50% [43].

One argument against metallic stent deployment is the interference with future surgery [43]. As we did in our patients, it is advisable to leave an unstented portal vein distal segment for surgical anastomosis in future LT [38].

The percutaneous transhepatic approach is the traditional method for portal vein catheterization and portal vein CTO treatment. The main advantage of transhepatic venography is that it allows accurate determination and extension of the obstruction, as well as direct portal venous pressure measurement which helps to establish the cause of portal vein CTO [44, 45].

Uller et al. [26] preferred the transplenic access, underlying many advantages such as the possibility to perform antegrade venography, a favorable way to cross tight portal vein occlusions especially in case of angled stenotic portal vein segments.

Because of the high vascularity of the spleen, the major technical issue is represented by the risk of bleeding, which can be effectively prevented with tract embolization during introducer sheath removal [15, 46, 47].

In line with our findings, several investigators independently reported that portal vein intervention via the transplenic and transhepatic accesses are both feasible and safe in LT recipients [15, 46, 47].

In portal vein interventions, minor complications such as postprocedural abdominal pain and fever are common and can be managed with medications. In addition, after TIPS creation, there is a theoretical risk of pulmonary embolism, that was never observed in our series. Hemoperitoneum is the most feared major complication of percutaneous transhepatic and transplenic portal venous interventions. In a study including 44 children, Pimpalwar et al. [48] reported a 27% bleeding rate with transplenic access. In our study major complications occurred in 2/15 (13%) patients, without sequelae. In the case of arterial complications, like the observed splenic artery perforation, interventional radiology offers effective tools for resolving the complication non-surgically, through transcatheter embolization. However, it is important to note that in the event of splenic vein or splenic capsule rupture, bleeding control may not be achievable with interventional radiology techniques alone. Therefore, we recommend that all such technically challenging procedures be performed under general anesthesia, with immediate access to resuscitation therapy and surgical intervention if necessary.

The main limitations of this study are represented by its retrospective nature and the small sample size, reflecting the rarity of the clinical condition.

## Conclusion

The findings of this retrospective study in conjunction with the few evidence in literature support PVR as a feasible, safe, and effective minimally invasive procedure with excellent technical and clinical success for the management of portal vein CTO in adult and liver-transplanted paediatric patients. The endovascular treatment may restore the native anatomy of the portal system, simultaneously offering the possibility to occlude porto-systemic collaterals; TIPS creation should not be the target, to be only considered on individual basis.

## Data Availability

The datasets used and/or analyzed during the current study are available from the corresponding author on reasonable request.

## Abbreviations

CIRSE: Cardiovascular and Interventional Radiology Society of Europe
CDUS: Color-Doppler Ultrasound
CTA: CT angiography
CTO: Chronic Total Occlusion
DSA: Digital Subtraction Angiography
IQR: Interquartile Range
IR: Interventional Radiology
LT: Liver Transplantation
MRA: MR angiography
PTA: Percutaneous Transluminal Angioplasty
PVR: Portal Vein Recanalization
TIPS: Transjugular Intrahepatic Porto-systemic Shunt
